# Abstracts and Gleanings

**Published:** 1877-09-20

**Authors:** 


### Abstracts and Gleanings.

DENTAL IRRITATION—*Bromide Potass. For.*—Charles W. Oleson, M. D., in a paper read before the Columbus Academy of Medicine, says: While many dispute the assertion that the irritation of the gums during dentition is at all liable to cause any disturbance of the general nutrition, or any sympathetic abnormal action of the alimentary canal, another class, with equal opportunities for observation, as confidently assert their opinion to the contrary. During the summer months cases are frequently seen in which attention having been previously given to the proper alimentation and thermometrical protection of the child, the vomiting, diarrhœa, and nervous disturbance still continue.

How can we account for this upon any other hypothesis, especially when we find the gums tense and livid, and suitable attention to them is rapidly followed by relief of the trouble. Instead of being a purely natural process, devoid of any injurious tendency, I believe it to be a prime factor in the genesis of these summer complaints.

To relieve this tendency, I have employed, with constantly increasing satisfaction during the last seven years, a treatment recommended by Dr. Caro in a paper read before the New York County Medical Society. While this treatment may be familiar to all, yet so important is it that I will read what he says upon this point, for if there is one of my hearers to-night to whom it is not known, the time will be well spent:

" In the most severe cases of odontitis, either with or without ulcerated gums and loosened bowels, I have never failed to relieve the child by the local application of brom. potass. Almost immediately after the first rubbing on the gums, from being turgid, swollen and red, they assume their natural color, and a certain amount of ease is felt. Saliva commences to dribble, and, as if by enchantment, agitation, carpo-pedal involuntary motion, vomiting, and looseness of the bowels disappear. As the vomiting and diarrhœa in this case are not the consequence of gastro-enteritis, but of an excitement of the stomach and intestinal mucous membrane, owing to the inflamed condition of the gums, I suppose it will never be cured either by the scarification of the gums, or by astringents or anodynes, as well as by brom. potass."

While I find this most satisfactory, I cannot agree with Dr. Caro as to the rapidity of the relief, and so in cases where there is no time to lose, I draw my gum lancet over the gum, cutting down to the tooth, and then give the bromide as supplementary treatment.

I have rapidly glanced at the agents most active in causing the departure from health in these serious and common complaints, and have directed attention to the measures applicable to their relief. Besides these measures I have resorted to medication but rarely, except in the case of one drug, the potass. bromide. This drug seems particularly adapted to the hyperæsthetic condition in children, and decidedly so when there is a tendency to irritation of the mucous membrane with increased secretion.

Different writers, commenting upon the bromide, give its action as follows:

Laborde says: " It exercises a predominant, and, to a certain degree, an elective, action on the nervous system, and especially upon the phenomena of reflex action."

Clark and Amory say: " It is a vascular and nervous sedative." " Its primary effect in passing out of the system is to diminish all the secretions save the urine."

Bell, United States Army, says: "It is an anæsthetic to the nerves of the mucous membrane, and a depressor of their action."

Bartholow recognizes "diminution of sensibility of mucous membrane, which he considers is in part due to a local action of the salt in beng eliminated."

So great has been my confidence in it as appropriate treatment to the conditions present in the outset of these diseases, that I administer it on their appearance from whatever cause, even going so far as to recommend my patrons to have a solution on hand, and upon the appearance of restlessness in the child order this sedative to be used. This is a solution of the bromide in syrup and water, so compounded that each teaspoonful represents one grain of the salt, the dose necessarily depending upon the age and susceptibility of the child.

As an adjunct to proper alimentation, protection from the effects of great heat, and supervision of the process of dentition, it acts as well as we can expect any one drug in any one condition.

TUBERCULOSIS—TRANSMISSIBLE.—A. N. Bell, M.D., in *Sanitarian*, says: "That tuberculosis is transmissible, is a fact well determined in late years by direct experiments; and this has, besides its scientific significance, a practical importance of such weight, that every contribution to the explanation of this, in many respects, obscure subject, is certainly most desirable. And of this class are the experiments in this veterinary school in relation to the transmissibility of tuberculosis."

Fleming, in his recent masterly work on Veterinary Sanitary Science, besides noticing the experiments of Professors Gerlach and Leisering, in the Veterinary Schools of Berlin and Dresden, mentions several other veterinarians who are similarly engaged, and nearly all show conclusive evidence of the infectiousness of tuberculosis by the digestive organs. Villemin, Klebs, and others have also successfully produced tuberculosis in rabbits, guinea-pigs and other animals, by inoculation with tuberculous matter, and in like manner demonstrated its contagiousness. Chauveau and Saint-Cyr, of the Veterinary School of Lyons, have done the same thing in calves and heifers. Harms and Gunther, of the Hanover Veterinary School, and Bagg, of the Copenhagen School, by feeding animals with the flesh and lungs of a tuberculous pig and a phthisical cow; Zurn, of Jena, in pigs, by first feeding them with the milk, and subsequently with the flesh of a phthisical cow. Viseur, of Arras, has been equally successful with cats. One of these animals, after death, was found to have all the lymphatic glands enormously enlarged, the mesenteric glands increased in size, and the lungs studded with white, hard tubercles. These cats ate the tuberculous matter voluntarily.

HYPODERMIC INJECTIONS OF MORPHINE IN THE REDUCTION OF INGUINAL HERNIAS.—Dr. Philippe, of Saint-Mande, recommends the use of hypodermic injections of morphine in recent cases of strangulated inguinal hernia, and reports three cases in which he administered them with excellent results. Taxis had been previously tried in vain, but after the injection of from one-third to one-half grain of morphine, it was resumed and proved entirely successful. One of the patients was an old man, ninety years of age, who was afflicted with a voluminous, irreducible inguinal hernia on the right side. During a paroxysm of coughing, a fresh loop of intestine was forced into the sac. The injections of morphia, however, can only prove serviceable at an early period after the descent of the intestine; at a later period they are far less valuable than anæsthetics, which not only relax spasm, but, if necessary, permit of an immediate resort to operation.—*Le Mouvement Medical.*

NOCTURNAL CRAMP.—A member writes: "I am very glad to find that J. E. C., M.D., has found some benefit from Howard's bicarbonate of soda. He has lain many nights studying cramp in his own person. It proceeds, he says, from excessive acidity, not only of the stomach, but of the whole bowel tract; and when it seems to have reached its height the extensor tendons have nearly dislocated the great toe. Then it is that relief is at once obtained by taking half a drachm to two drachms of the soda. Before he found this remedy useful, many things had been tried. In less than thirty seconds the cramp disappears, leaving a soreness that soon passes away. It has been prescribed by him in numerous cases, and the result has been always satisfactory.—*Brit. Med. Jour.*

PROLAPSUS RECTI.—This is a rare condition among children. It is of varying grades, as of part of the mucous membrane, or the whole of the rectum up to the sigmoid flexure. The latter is usually after the former has been allowed to pass unnoticed for a long time. In most cases, however, we find only a partial prolapse occuring after constipation. Catarrh of the large intestine may be a cause of prolapse, by the frequent stools and the tenesmus occurring coincidentally with the wasting of the muscular part of the intestine. In rachitic children with such a catarrh, it not infrequently occurs disappears for awhile, and reappears with the exacerbation of the catarrh. Such cases are best treated by treating the intestinal catarrh, and by irrigation of the intestine with water, beginning with a temperature of 24° to 22° (C.), and descending to that of fresh spring water.

In chronic cases, astringent irrigation with solutions of alum and tannin should be used.

Such are also benefitted by local treatment with cauterants. The prolapsed bowel may be lightly touched with nitrate of silver in substance, making a circle round it and radiating lines along the axis of the intestine; after this it should be replaced and confined with a suitable bandage. This should be renewed every three days for three or four weeks. If such proceedings do not effect a cure, one should use the hot iron, especially when the prolapse has lasted long and the sphincter ani is paralyzed. The irons used used should be small, and applied at the line where the mucous membrane covers the common sphincter. Strychnia and nux vomica by hypodermic injection, or suppository, he does not think of much value.

The replacement of a prolapsed rectum requires care. If a child is alarmed and screams and strains, it is best to anæsthetize him first. One must not maltreat the intestine with futile manipulations. When the intestine is replaced, it should be secured with a retainer of some sort. Dr. Monti uses, and thinks better than any of the more complicated appliances, a series of strips of adhesive plaster, which cross over the mons veneris and anus, constituting a sort of artificial sphincter. Through the part opposite the anus he cuts a hole, through which the stools pass quite well, and yet the application prevents the protrusion of the rectum.—*Phil. Med. Times.*, Aug. 4, 1877.

GUARANA IN MIGRAINE.—A correspondent writes to the *British Medical Journal*: Having used guarana in a great many cases, I have come to the following conclusions:

1. True migraine, characterized by acute frontal pain, commencing on one side, occasionally both, or going from one side to the other, usually lasting from twenty-four to forty-eight hours, with or without sickness, and relieved or cured by sleep, whether caused by errors in diet or not, will almost invariably yield to it.

2. In young persons, in whom the habit is only commencing, not only does it cure each individual attack, but, by persevering, the habit itself is broken.

3. One cause of failure is the smallness of the dose, so that, in many cases in which it has been tried before and failed, an increase of the dose has been followed by cure. Twenty-five grains of the powder is my usual dose for an adult female, half a drachm for a man; less, of course, for younger cases, repeating in one or two hours, if necessary.—*Louis. Med. News.*

Tong Pang Chong.—Dr. Murray, in *British Med. Jour.*, says of this Chinese remedy:

I have submitted some of the root, through the kindness of my friend Dr. Hooker, to Mr. Jackson, the curator of the Kew Museum, who pronounces it to be "the produce of a Berberideous plant, and nearly identical with *Akebia quinata* (Decaisne)." Mr. John Thomson of King's College has extracted from the tincture a crystalline substance, which may prove to be allied to chrysophanic acid, and so account for its action.

I shall not pretend to enter into any explanation of the *rationale* of its curative power, but limit myself to the statement that I have used it in hundreds of cases, often with almost magical effect, and that it is now much employed in the East. It is necessary, however, to discriminate in selecting the cases in which to test its value. I have found it most useful in tinea circinata, where the circular margin of the disease is maintained; and perhaps it is even more successfully used in that very troublesome form of eczema (tinea?), which attacks the inside of the thighs and perineum, and the parts around the anus, which I have known to resist every description of treatment, internal and external, even in the most experienced hands; and I may add that both in this and other forms of disease, I have found it succeed where the Goa powder had completely failed.

It is used in the form of a tincture prepared by mascerating the root in the impure native spirit called *arrack.* May be prepared with alcohol as well.

The method of application which I follow is to paint the part over by means of a camel's-hair pencil three times, allowing it to dry between each coat, and this is done every night at bedtime, until the part resumes its natural appearance.

*Some Remarks on the Introduction of the Whole Hand into the Rectum*, is the title of a valuable paper by Mr. W. J. Walsham. The author makes the following remarks, deduced from the examination of four cases on the living body and twelve experiments on dead bodies:

1. "That the hand, if small, can be introduced into the rectum of both male and female without fear of rupture of the sphincter or incontinence of feces.

2. "That the dilatation of the sphincter should be very gradual, five minutes at least being allowed for its accomplishment.

3. "That no pain or inconvenience is experienced by the patient as an after-result of the operation.

4. "That when once through the sphincter, the windings of the gut should be followed very cautiously by a semi-rotatory movement of the hand, and by alternate semi-flexing and extending the fingers.

5. "That in many cases the hand can be passed into the sigmoid flexure, and possibly, in rare instances, into the descending colon.

6. "That should the hand meet with a feeling of constriction about the junction of the first and second pieces of the rectum, no force on any account should be used to overcome it, as this can only be accomplished by rupturing the peritoneum, which is here reflected from the intestine.

7. "That this method of investigation is of use in detecting a stricture high up the rectum or in the sigmoid flexure of the colon, but that a stricture below the descending colon may exist although the hand may be unable to discover it.

We believe Mr. Walsham's investigations on the point he has chosen for his paper calculated to do a great deal of good in throwing light on some obscure pelvic maladies.—*Amer. Jour. Medical Sciences.*

The Poison of Small-Pox.—In the session of the French Academy of April 30th, Messrs. Pasteur and Joubert made an important communication on the na ture of the poison of small-pox (hæmorrhagic variety). They have succeeded in propagating the bacteria, which are contained in the blood during this disease, outside of the living organism, in "dead" liquids; and they found that their vitality remained unimpaired, even after repeated transplantation. Such an infected solution may be filtered by means of appropriate apparatus, and completely freed from bacteria, so as to become entirely inert. During the artificial propagation of these bacteria in clear solutions, no microscopic granulations appear to be formed, at least even under the most powerful lens, no organized or amorphous substances can be traced outside of the bacteria. These facts make it highly probable that the poison of small-pox is a bacterion, and not a virus.—*Ber. d. Deutsch. Ch. G.*, 1877, 1171.

EFFECTS OF PILOCARPINE USED HYPODERMICALLY.—Frohnmuller finds that crude pilocarpine, a reddish-brown extract of weak alkaline reaction, imperfectly soluble in water, easily dissolved in alcohol, in pill form, 0.016 to 0.02. grm. ($\frac{1}{4}$ to $\frac{1}{3}$ grain), produces the same effect as an infusion of 5 grammes (80 grains) of jaborandi leaves, even in relation to the unpleasant after-effects. The muriate of pilocarpine, a crystalline substance of acid reaction, easily soluble in water, is, according to F., very well adapted to subcutaneous injections. He injected $\frac{1}{5}$ to $\frac{1}{4}$ grain, and observed after a few minutes the well known effects of jaborandi, profuse perspiration and salivation, disturbance of vision, particularly for near objects, nausea, and even slight vomiting, and finally transitory increase of frequency in pulse and respiration.

PITYRIASIS CAPITIS.—This disease has been cured by chloral, which not only calms the itching, but destroys the morbid epithelial production which constitutes the pellicle of the pityriasis. It has also been found beneficial in the itching of exzema, in vulvar pruritis, and other pruriginous maladies. The parts affected should be bathed or lightly rubbed two or three times daily, with a mixture of hydrate of chloral one part, dissolved in twenty parts of distilled water; it should not be dried with a towel. The application causes a slight heat and redness of the skin for a minute or two, and should be continued daily for a month or two, according to the obstinacy and chronicity of the malady. *Dujardin-Beaumetz, L. Martineau.*

JABORANDI IN ASTHMA.—Dr. Gubler succeeded in five cases in aborting the attack by giving an infusion of the leaves, relief being obtained as soon as its sialogogue and sudorific effect appeared. He found the jaborandi to produce instantaneous amelioration of the asthmatic paroxysm of emphysema. To one man a cup of tepid infusion was administered during an excessive paroxysm of asthma, who fifteen minutes afterward began sweating and expectorating. Almost immediately after this the respiration became easy, the patient declaring that the malady had been taken from him as with the hand.—*Materia Medica.*

BENZOATE LITHIUM IN GOUT.—The treatment of gout by benzoate of lithium is recommended by Trehyon, in the *Rev. de Therap. Med.-Chir.* This salt is unlike the other lithium compounds, easily soluble in water; furthermore, the benzoic acid, being converted into hippuric acid, diminishes the elimination of uric acid. Instead of the insoluble urates, easily soluble hippurates are formed, which are excreted with the urine. These theoretical considerations have been verified by experience. During the continued use of benzoates of lithium, the gouty attacks become milder and less frequent, and the osteal pains disappear.—*Memorabilien*, July, 1877.

GASTRO-ELYTROTOMY.—This novel and singular operation, referred to by Dr. Thomas in his late work, is thus described by Dr. E. W. Jenks in a paper read before the Detroit Medical Association:

"*Gastro-elytrotomy* is so new a procedure that statistics concerning it are exceedingly limited, but theoretically it is a simple operation and promises far more in its results than the Cæsarean section or even cephalotripsy. It consists in making an incision just above and parallel with Poupart's ligament from the symphysis pubis to the anterior superior spine of the ilium, holding back the peritoneum as in the operation for ligature of the iliac arteries, opening the vagina at its junction with the cervix, and extracting the child by version."

To Relieve Morbid Thirst for Alcoholic Drink.—S. B. Merkel, M.D., of Philadelphia, writes to the *Journal of Materia Medica* as follows:

"A tonic and stimulant which partially supplies the place of the accustomed liquor, and prevents the absolute moral and physical prostration that follows a sudden breaking off from the habitual use of stimulating drinks:

R. Peppermint water........oz. xij.
Sulphate of iron.........gr. v.
Spirits of nutmeg........oz. ij.
Valerianate of quinia....gr. ijss.

S. Teaspoonful taken as often as the desire for strong drink returns. I have had frequent occasion to test its efficacy in many cases in my practice, and have found it uniformly successful.—*Louisville Med. News.*

Simple Mode of Checking Epistaxis.—The *Tribune Medicale* says that even after plugging the nares, injection of perchloride of iron, etc., have failed, an emetic, given to the extent of producing vomiting, will permanently check epistaxis—*Ib.*

Protection Against Flies — For Doctors' Horses.—

R. Linseed Oil................dr. xij.
Carbolic acid crystals...oz. ij.
Glycerine................dr. jss.

Dissolve the glycerine and add the oil. Apply daily to legs, mane, tail, face, neck and flanks; and the flies are driven off, much to the delight of the horses.—*Canada Lancet.*

A New Preparation of Iodine.—Mr. J. Crouch Christopher calls attention to a new preparation, which, he says, in his hands has proved to be more useful and to have fewer disadvantages than any other remedies of like nature in more general use. It consists of twelve grains of cinchona flava, one grain and a half of iodine in the form of hydriodic acid, and one grain of protoxide of iron to a fluid drachm of liquor. The fact that the iron compound remains in the state of proto-salt (whereby its value is enhanced), and that the liquor never, either by time or exposure, becomes inky through the action of the tannin in the bark, tends to show that there is something more in this case than a mere mixing of ingredients.

The cases in which this preparation or compound has been found most useful were, for the most part, cases of secondary and tertiary syphilis, particularly those in which mercury had been lavishly used or abused—cases in which it was difficult to determine to what extent the diseased condition was due to syphilis, to the abuse of mercury, or to a combination of both. Great benefit has been derived from its employment in cases of persistent and frequently recurring boils, at a time when what may be termed a furunculoid epidemic existed.

It has been serviceable also in cases wherein it was important to give iodine in some form without incurring the risk of depressing the patient unnecessarily—such as cases of scrofula, anæmia, and glandular enlargement. Some of these, intolerant of the officinal preparation of iodine, tolerated this, and were benefitted by it.—*Phila. Med. Times.*

The Subcutaneous Injection of Morphia with Atropia.—Dr. Lagoda (*St. Petersburg Med. Wochenschr.*) employed the combined injection of morphia and atropine in two exquisitely painful cases of neuralgia, in one of which morphia given alone produced severe vomiting, headache and loss of appetite. Other anodynes and narcotics were of no decided benefit. One-twelfth grain of morphia with one-fiftieth grain of atropine was then injected subcutaneously. Soon thereafter the pain ceased, quiet sleep set in, and on the following day the patient was free from nausea and headache; the appetite remained good. These injections were continued at weekly intervals in the same doses and always with a similar effect. Atropia injected by itself remained without effect.—*The Clinic.*

Nux Vomica in Opium-Poisoning.—Dr. H. H. Beck, of Sulphur Springs, Texas, writes to the *Louisville Medical News*:

On Sunday night, the 29th July, at eleven o'clock, visited a child aged eighteen months, who, at six o'clock, the mother gave a grain and a half of sulph. morphia by mistake for quinine. I found the little patient with every symptom in its worst form from the drug. The pupils contracted to immovability; pulse not perceptible at the wrists. I did not time respiration, but it was certainly much slower than I ever before witnessed.

I used with the little patient stimulants, counter-irritation, strong coffee, in fact all the usual means in such cases, without any apparent effect. It being five miles in the country, I had to send to my office [illegible] the tincture of nux vomica, which arrived at precisely two o'clock A.M., at which time the little sufferer looked as if done with the things of earth. I immediately, on arrival of the tincture, proceeded by giving hyperdermically, eight drops of the officinal tinct. of nux vomica. Its action was more like magic than physic. In the space of three minutes the little one opened its eyes, and pushed my hand off of its arm. In the course of one hour's time I could see the effects of the morphine beginning to preponderate. I again gave eight drops more, and by eight o'clock A.M. I had given in all thirty-two drops (hyperdermically) of the off. tinct. nux vomica, the little one going on to recovery without an untoward symptom.

I was induced to try the tincture from seeing a case reported. Can unhesitatingly say to the profession they need have no terror from opium-poisoning in future.

The Rash Produced by Bromide of Potassium.—At a late society meeting in London, Dr. David Lees exhibited an infant, aged nine months, with a pronounced bromide rash. A month ago it had convulsions, and had five grains and a half of bromide of potassium given every three hours, under the opinion that it was suffering from acute hydrocephalus. Under this treatment the symptoms quite disappeared. The rash appeared in spots, like the points of acne, with a yellow spot in the centre. These spots then coalesced and formed patches; these patches again became covered with a crust, which was really a dried secretion. Where these crusts were exposed to friction, as on the neck, long papillæ might be seen. These minute yellow spots were characteristic. In this case the rash came on as the bromide was being given up. The president remarked that it was curious how these rashes were produced in some persons, while others could take any amount of the bromide without any such result.—*Med. and Surg. Reporter.*

Retention of Urine.—Dr. F. Fischer, (Madison, Wis.,) relates to *Med. Brief*, the following method of relieving a case of retention of urine:

"At the time of my examination retention had lasted 48 hours, the bladder was distended fully to the umbilicus, pain was excruciating, especially at intervals during the efforts at contraction of the bladder. The pulse was somewhat accelerated, but not small; no symptoms of uræmia or peritonitis, but of considerable extension from pain and restlessness. Of course the inevitable indication was to empty the bladder. A number 6 catheter went in quite easy; but no urine was drawn. On withdrawing and examining it, it was found to be filled with coagula. A number 10 catheter was then used, which did not enter as readily, owing to an enlargement of the prostrate; but, although it was introduced up to its end, and the abdomen compressed with the palm of the hand, not a drop of urine would flow. I then used the kind of aspirator that I had been using at times years ago, when, on puncturing abscesses or cysts with the fraikart, the canula would become plugged—namely, *the rubber bulb* of a common injection pump. I fastened the bulb previously compressed by hand to the end of the catheter, and gradually relieving it, found it filled. When removed, the catheter would not drain, owing to a coagulum, which protruded from it several inches long; but the bulb was filled with coagulated blood. The bulb was then emptied, and reapplied four times with the same result; but after that time, a full stream of bloody urine, of a very offensive odor, was flowing, and over a gallon of it discharged, to the great relief and blessing of the patient.

The diagnosis in this case seems to me to be a little questionable. The patient, who was 72 years old, but still remarkably robust, had been subject to retention at previous times, probably owing to enlarged prostrate. But what had caused the hemorrhage, whether ulceration of the bladder or injury by preceding attempts at catheterism, or rupture of blood vessels by over-extension, I think is mere conjecture. The subsequent development of the case did not throw any more light on it. For two weeks, a limited hemorrhage with an actual retention of urine took place, which made the above described process necessary every day. During the next two weeks the catheter alone would drain the bladder; but still there was some admixture of blood to the urine; after that time, the urine became clear, and gradually the muscular action overcame the difficulty, so that patient, at the end of two weeks, was entirely restored, and is now enjoying good health. In this case, I suppose, if puncture had been resorted to, probably the same difficulty in removing the contents of the bladder would have been experienced besides the consequences of so serious a lesion."

Taynya, a New Remedy for Syphilis and Scrofula.—The latest medical journals announce the discovery of a new remedy—the so-called Taynya—in the treatment of syphilis and scrofula. From a report made before the Congress of Physicians at Turin, is the following abstract:

This agent, which is now in possession of the brothers Ubicini at Pavia, was found by one of them in a journey through Brazil, to have been used by the inhabitants of this land in the treatment of syphilis with success. He forwarded the roots of the plant to his brother in Pavia, who sold it to physicians, hospitals, and pharmaceutists, at the rate of 30.0 grm. for four marks.

The plant grows in wild forests and on mountains, in stony regions, and often among coffee plants. All parts of it are efficacious, but the roots are preferable, and two alcoholic tinctures are prepared from them. The stronger of these tinctures—the tinctura madre—is used hypodermically, in gramme doses, and diluted with water as compresses and applications. For cataplasms, a decoction of the root is made. The weaker tincture—the tinctura diluta—consists of one part tinctura madre with three parts rectified spirits, and is given internally in doses of 2-20 drops two or three times a day.—*Clinic.*

Hydrophobia Cured by a Rattlesnake Bite.—At the annual meeting of the Missouri Institute of Homeopathy held in May last, the proceedings of which are printed in the *Medical Investigator* of Chicago, a new remedy for hydrophobia was proposed as follows: "Dr. Philo G. Valentine, of St. Louis, chairman of clinical medicine, read several papers of interest, one in particular, where hydrophobia was cured by the bite of a rattlesnake. A man had been bitten by a rabid dog, and when he felt the symptoms of hydrophobia coming on he went to his family and told them that he must leave them, and they must shut the house and not let him in, as he might injure some member of the family. He accordingly went out, and came to a spring of water, the sight of which threw him into convulsions, and while writhing on the ground was bitten by a rattlesnake, and almost instantly cured, and never troubled by the poison thereafter." [?]—*Pacific Med. Jour.*

REMEDY FOR HOOPING-COUGH.—(*Lyon Medical* No. 11, 1877.) M. Dervieux believes he has found a preservative means in aconite, associated with ipecac and cherry laurel water. This mixture is either a veritable preventive, or simply an abortive. His formula is as follows:

| | | |
|---|---|---|
| Extract of aconite, | .05 grammes | —$\frac{4}{5}$ gr. nearly. |
| Cherry laurel water, | 4. " | —1 dr. " |
| Syrup of ipecac,.... | 3. " | —$\frac{3}{4}$ dr. " |
| Mucilage.......... | 200. " | —$6\frac{1}{2}$ oz. " |

This is given as soon as the characteristic cough presents itself, in doses of a teaspoonful every hour, to young infants; two teaspoonsful to those more than three years of age, and a teaspoonful to adults every hour.—*Chicago Med. Jour. and Ex.*

A MEANS OF ARRESTING HOOPING-COUGH.—Lansinski recommends the insufflation into the larynx of the patient of a small quantity of a powder composed of 30 grains of salicylic aid, 15 grains quinine, 7 grains of bicarbonate of soda, and 7 grains of sugar. This should be done twice daily, and the above quantity should last about ten days. He adopted this treatment in fifteen cases of severe hooping-cough, and were cured in periods varying from eight to thirty days.—(*Deutsch. Med. Wochens.*)—*The Practitioner.*

Albuminate of iron, as a remedy in anæmia and chlorosis, deserves to be better known. It is soluble and more readily absorbed than any other organic or inorganic compound of iron. Particularly good results have been obtained from its use in anæmic women.—*Dr. Choisnard,* in *Gaz. des Hopitaux,* June, 1877.

PATHOLOGY OF URÆMIA AND THE SO-CALLED URÆMIC CONVULSIONS.—Dr. F. A. Mahomed, in the *British Medical Journal* for July, advances a new and very plausible theory to account for the convulsions of uræmia. In Bright's disease there is always a condition of high arterial tension, together with changes in the heart and arteries generally. As a result of these, various hemorrhages may occur from rupture of minute or larger blood-vessels; these of en take place from or into the mucous membranes of the nose, intestinal tract, uterus, and, perhaps, air-passages and bladder. They are also known to occur below the serous membranes, as in the peritoneum, pleura, or pericardium. They are frequently seen in the retina, preceeding or accompanying "albuminuric retinitis." They are very commonly seen in the brain, causing extensive hemorrhage; it is, indeed, the most common cause of apoplexy. Small capillary hemorrhages may, and do very frequently, occur in the brain as the result of Bright's disease, and these hemorrhages are the causes of the epileptiform convulsions generally known as uræmic, and all the so-called uræmic symptoms are due to the results of high tension on the capillaries of the brain, producing rupture of, or exudation from, them. The reason why this was not previously commonly observed is, that such hemorrhages usually occur in the grey matter of the convolutions, and that it is manifestly impossible that all the convolutions of the brain should be examined for minute punetiform hemorrhages, whose very existence was not even suspected. Several cases are reported in detail where the evidences of both recent and old hemorrhages seemed to have been the cause of the epileptiform convulsions prior to death.

While bearing in mind the miliary aneurisms described by Charcot and Bouchard, and also the similar true and dissecting aneurisms of the small cerebral vessels (especially those of the cortex) described by Rindfleisch and many others, and the well known forms of degeneration affecting the smaller vessels and capillaries of the brain, often seen in connection with Bright's disease and other allied conditions, and that with these anatomical changes frequent variations in the arterial tension occur, it will be admitted that there exists in Bright's disease abundant cause for the occurrence of all forms of cerebral hemorrhages: moreover, these eschymotic spots are true hemorrhages, and not merely the sacs of minute aneurisms transversely divided.—*N. O. Medical & Surgical Journal.*

Mercurial Vapor Bath.—Prolonged and obstinate cases of syphilis may frequently be cured by the use of the mercurial vapor bath. A very simple method of using this bath is that recommended by Mr. Henry Lee.

A tin case is used, containing a spirit lamp, and having in the centre, over the flame, a small tin plate, on which 15 to 30 grames of calomel are placed, and around this a sort of saucer filled with boiling water. The lamp being lighted, the apparatus is placed under a common cane-bottom chair, on which the patient sits, enveloped, chair and all, in one or more large blankets for about twenty minutes, when the water and mercury will be found to have disappeared. It is better not to use a towel, as the calomel would be wiped off by it.–*Dunglison.*

Tar Water in Typhoid Fever.—Fresh tar, prepared by burning rich pine under a pot, constitutes the best terebinthinate in typhoid fever, as it is more pleasant than the oil of turpentine, acts gently upon the kidneys, without producing strangury; contains a little creasote, and is antiseptic in its properties, tending to destroy germs and eradicate the typhoid poison from the system. We have used it much in practice and with satisfactory results. Our method is the following:

To one tablespoonful of the tar pour a pint of water. To be taken during the day; the same to be freshly prepared every morning and the air excluded by covering the vessel. We have been in the habit of recommending it as prophylactic to the disease, and we believe it to be efficacious in this regard.

W.

The Hypophosphites in Phthisis.—*Case* 1.—James R. E. was an out-patient at Victoria Park Hospital, May 9, 1857. He was a pale, thin young man; had been ill, with more or less cough, for the last five years. He dated his illness from a sudden spitting of blood. The left side of his chest was flattened, with impaired percussion resonance and abundant crepitant *rales* in inspiration. The right side of the chest was resonant; expiration was prolonged. Cod liver oil always made him sick. On the previous day he brought up blood. He was ordered to take five grains of hypophosphite of soda in camphor-water three times daily. May 16th the medicine agreed well, and he felt much better. On May 23d the cough was much better. Pulse 104. There was a cooing sound with expiration in the right lung. The left side was dull at the upper part, and here a dry creaking was replacing the crepitant *rale.* He was ordered to take five grains of hypophosphite of lime in place of the soda salt. On May 30th he was much amended; there was very little sputum now. On June 13th he felt himself well, though respiration was not normal in the left lung. He could now take some cod liver oil, and, at his own desire, left to go to his home in Wales.

*Case* 2.—Benjamin D., a laborer, aged about 35, from Acton, was seen on June 27, 1867. He had had a bad cough since March, with frequent spitting of blood. Pulse 104, feeble. The bowels were inclined to diarrhœa. The tongue was clammy. His breath was very short. Both sides of the chest were somewhat flattened. The respiratory sound was generally weak. Crepitant *rales*, to a slight extent, were heard over the left upper third. The liver was enlarged and tender. Cod liver oil, he said, "always ran through him." He was ordered to take five grains of hypophosphite of lime with ten minims of saccharated solution of lime in infusion of calumba three times daily. He took this till August 8th, when he was discharged, stating that he could now walk a long distance without fatigue; his cough also was "nothing worth speaking of." Dry, creaking noises could be heard still at the upper part of the left lung.

CHOREA.—Dr. Arnold. A young lady, aged eighteen, had suffered with chorea for two years and a half. Nearly every known remedy had been tried without avail. When I saw her every voluntary muscle seemed to be affected; could hardly talk, and had to be fed with a spoon. Menstruation was regular, the countenance was pallid, great anæmia, and a basic systolic murmur which is common in bad cases of chorea. I ordered perfect rest, put her in a crib and strapped her body so as to restrain the convulsive movements. Three times a day she had alternate hot and cold douches to the spine, continued as long as she could endure them, and iron internally. In two weeks there was marked improvement, and in five weeks she was so far recovered as to be able to go shopping, and if not hurried you would notice nothing amiss. If hurried she shows some little excitement and stammers, but this subsides in a few minutes.

Dr. Rennolds. I attended a girl fourteen years old, who had had chorea for two years. I gave her calomel and jalap, each ten grains, repeated daily, and she was well in two weeks. Some years ago I read a paper before this society, giving a number of cases relieved by this treatment in a very short time.

Drs. Winternitz and Seldner expressed themselves as having tried the calomel and jalap treatment with failure in every instance.—*Med. and Surg. Rep.*

GONALGIA, OR HYSTERICAL ARTHRALGIA.—The peculiar affection known as "knee-ache," is not uncommon among women of relaxed fibre, and in conditions of general debility, in both sexes. It is usually regarded as a form of rheumatic gout or neuralgia; but I have found it as frequently associated with uterine disorders and hysteria as in cases of gouty or rheumatic diathesis.

In some cases the knee only is affected, in others a dull, diffused, aching pain occurs in the wrists, soles of the feet, ankles, heels, and toes. Sometimes the pain is paroxsymal, coming on suddenly during the day or night, lasting a few hours or several days, and is usually accompanied with more or less pain on movement, and tenderness on pressure.

The patient often complains of the "knee giving way," and of a "cracking sensation" in the joint, and persons have even resorted to mechanical appliances, under the impression that the knee needed support. Occasionally the joint is found pale, puffy, or swollen, and there may be hyperæmia and hyperæsthesia, with change of temperature, and a tendency to cramp of the sural muscles.

In women who have borne children rapidly, and whose nervous systems have been broken down, the left leg or knee is most commonly affected, and there is a strong tendency to spasm of the muscles of the lower extremities, with involuntary contraction of the toes, and a peculiar nervous twitching, especially noticeable during sleep. In nearly all these cases, anti-neuralgic remedies alone have but little permanent effect, while flying blisters, stimulating embrocations, and lotions are almost useless. The treatment that promises most speedy relief is milk diet, the mineral tonics (with small doses of colchicum in gouty cases,) change of air and scene, douching the painful joint alternately with hot and cold salt or sea water; the use of Chapman's "spinal bags," and last, but not least, covering the affected part with a sheet of thin rubber.

The rapid effect of the latter in some cases, is remarkable.

If the knee-joint is affected, a close-fitting knee-cap of sheet rubber should be worn at night, and may be removed in the morning, if all pain and weakness has disappeared, or it may be kept on until it induces a decided eruption, or irritation of the skin, when it should be removed and the part washed with alcohol or bay rum. The prompt and efficient relief which the sheet rubber has given in several cases of gonalgia which have come under my notice, justifies a further trial of its merits in this painful and annoying affection.—*C. J. Cleborne, M.D., in Med. Record.*

WHAT IS CHRYSAROBIN?—Chrysarobin is the term which has been selected to designate what is more commonly known —because so first known—as Goa powder. It has been chosen for these reasons: That Goa powder is so called only because it is derived by the rest of India from the port named Goa; that the same powder is known over South America as Bahia powder, except in the province of that name, from which the other parts of the country receive it; while in that province it is known by its native name of Aroba-powder. But since this powder is the active part of a whole tree, rather than continue the compound word aroba-powder, it is convenient to substitute the single word arobin. Yet further, while Goa powder (or old powder) is brown, Aroba powder (a newly prepared powder) is yellow. Yellow is the right color. Hence, to arobin is added the prefix chrys, and thence is formed chrys arobin—*i. e.*, yellow Aroba powder.

The action of chrysarobin is emetic and purgative. Vomiting is always the first sign of action. This is not attended by any depression at all compared with that caused by tartar emetic or ipecacuanha. In the doses presently to be named, it has not caused any distressing retching; and in children, as well as in adults, the acts of vomiting varied between none in three out of the whole number, and six in two out of the whole number. They were usually two or three; very often only one. The action on the bowels was much more variable, from none in a few cases to nine or ten in equally few cases; most often the range was between three and seven. There is no griping pain, but the nausea continues more or less markedly until the bowels recover. The motions are very watery, and of such a brown color as suggests its origin with the powder taken. If the vomiting be very early, then the purgation, although marked by a fluid stool or stools, will certainly not be violent; and in some cases, in which there was no vomiting, the bowels acted very freely. It does not always happen so, however, under the same condition; and I conclude, therefore, thst some persons can take a larger dose than others. I cannot distinguish these persons any more than I can accurately guage the amount of any other purgative which a given person will require at first seeing him. If the dose be taken into a full stomach, that delays its action and determines it to the bowels.—*Druggists' Circular.*

HYDROBROMIC ACID IN TINNITUS AURIUM.—The following case, selected out of several, of the successful treatment of long standing tinnitus aurium by hydro bromic acid, well illustrates the principles laid down by Dr. Woakes, in the *Journal* of June 23d. It will be seen that in this case the tinnitus was of the knocking or pulsating kind, and therefore probably due to a congested condition of the labyrinthine blood-vessels. In other cases, in which the tinnitus was of a continuous roaring or rushing character, the administration of hydrobromic acid had no beneficial effect.

I. S., aged 34, applied at the Central London Throat and Ear Hospital on May 11th. He had been deaf, and had loud "thumping" noises in the head for twelve years. There was no history of otorrhœa. On examination, the meatus was fairly healthy; hearing power was extremely defective; voice was only heard when much raised, and the watch not at all; the tuning-fork was heard on the mastoid process. The tinnitus was complained of as the most distressing symptom. He was ordered benzole inhalations, and hydrobromic acid in fifteen minim doses three times daily. On June 4th he reported that the noise had quite stopped, and said that his ears "felt much clearer and more healthy since taking the medicine." June 11th there was no return of tinnitus.—*British Medical Journal*, July 7th.

ACUTE AMMONIACAL COLLAPSE. (*Cent.albl. f. Chir., p.* 694, 1876. *Med. Times*, Jan. 20, 1877).—W. Roser, from a series of cases of acute ammoniacal collapse, draws the following conclusions:

1. Acute ammoniæmia arising from the absorption of decomposing urine excites sudden collapse, with decided lowering of the temperature of the blood.

2. In cystitis and pyelitis this lowering of the temperature may serve as a diagnostic sign of ammoniæmia.

3. If the originating cause of the acute ammoniærnia can be removed without loss of time, for instance, by puncture of the bladder, urethra or pelvis of the kidney, the collapse is frequently observed to pass off.

4. Ammoniæmia deserves much more attention at the hands of surgeons than it has hitherto received.—*Detroit Medical Journal.*

THE IMPORTANCE OF CINCHO-QUININE AS A REMEDY.—The Supervising General of the Marine Hospital Service has issued a circular letter to the medical officers of that branch of the Treasury, in which he calls attention to the extraordinary increase in the market price of sulphate of quinia, and at the same time alludes to the success attending the employment of the other alkaloids of the bark.

In the year 1866, the Madras Government appointed a Medical Commission to test the respective efficacy, in the treatment of fevers, of quinine, quinidine, cinchonine, and cinchonidine, and the remedial value of these four alkaloids, as deduced from their experience, is shown as follows:

| | | |
|---|---|---|
| Quinidine, | ratio of failure per 1000 cases, | 6 |
| Cinchonidine, | " " " " " " | 10 |
| Quinine, | " " " " " " | 7 |
| Cinchonine, | " " " " " " | 23 |

Cincho-quinine contains all these alkaloids, and the combination has proved more efficacious than any one alone; and the price of this article being less than one half the price of sulphate of quinine, the physicians of the country are substituting it for the sulphate; and the medical officers of the Government service should give this subject due consideration in preparing their requisitions for medical supplies.—*Washington, D. C., Daily Nation, August* 8, 1877.

